# Quantitative Determination of Pravastatin in Pharmaceutical Dosage Forms by High-Performance Liquid Chromatography with Ultraviolet Detection

**Published:** 2008-06

**Authors:** Safwan Ashour, Husni Nakshbandi, Soulafa Omar

**Affiliations:** *Department of Chemistry, Faculty of Sciences, University of Aleppo, Aleppo, Syria*

**Keywords:** pravastatin, liquid chromatography, pharmaceutical dosage forms

## Abstract

A simple, sensitive and precise high-performance liquid chromatographic formulations assay for pravastatin (PVS) is described. Good chromatographic separation was achieved using a Teknokroma C8 (5 μm, 25cm × 4.6mm) column and a mobile phase consisting of 10m*M* ammonium acetate: methanol: triethylamine (40:60:0.17 v/v/v) while at a flow-rate of 1.0 mL min^-1^. PVS was detected at 239 nm and was eluted 2.15 min after injection. No endogenous substances were found to interfere. No internal standard was required. Linearity range for PVS was 0.4-1000 μg mL^-1^. The determination of intra- and inter-day precision (RSD) was less than 2.94 and 2.97%, at all concentration levels. Statistical treatment of the experimental results indicates that the method is precise and accurate. The proposed method was applied to the determination of the component in commercial tablets with no interference from the excipients. A comparative study between the suggested procedure and the pharmacopoeial method for this compound in the tablets showed no significant difference between the two methods.

## INTRODUCTION

**Figure 1:**
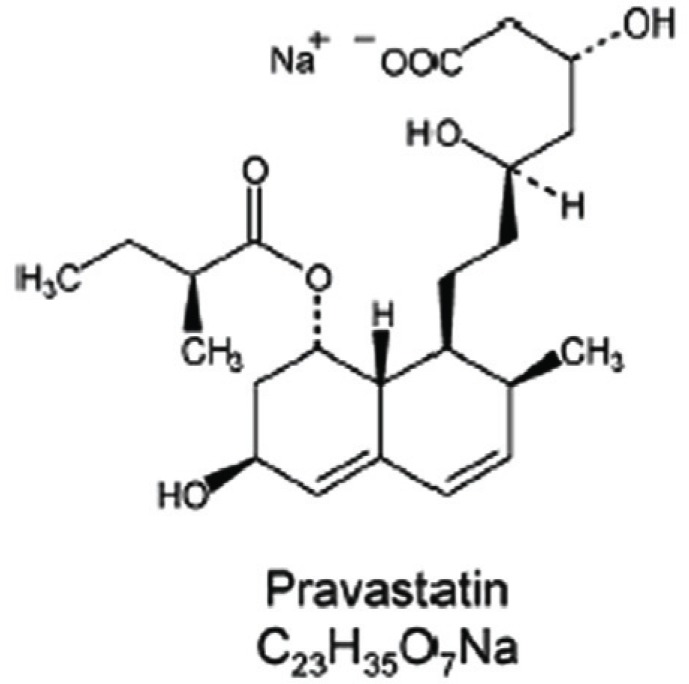
The chemical structure of pravastatin (sodium salt).

Pravastatin (PVS, Fig. 1), hexahydro-6-hydroxy-2-methyl-8-(2-methylbutyryloxy)-1-naphthyl-3,5-dihydroxyheptanoate, is one of a class of lipid-lowering compounds, the HMG-CoA reductase inhibitors, which reduce cholesterol biosynthesis. These compounds are used for the treatment of hypercholesterolemia. Pravastatin is characterized as one of the best, due to the hydroxyl group attached to its decalin ring, which results in a greater hydrophilicity than other HMG-CoA reductase inhibitors (1-3). Few methods for the determination of PVS in pharmaceutical preparations have been reported in the literatures. High performance liquid chromatographic and capillary electrophoresis methods were developed to determine PVS in production media. The analyses were performed on particle column, monolithic column and silica capillary filled with borate buffer pH 9.3 containing 20 mM SDS (4). PVS was determined using liquid chromatography–electrospray ionization tandem mass spectrometry with methylammonium acetate as an additive in the mobile phase. Protonated PVS was selected as precursor ion, and product ion was detected by selected reaction monitoring in positive-ion mode (5). A capillary electrophoretic method using a fused silica for the determination of PVS in pharmaceutical tablet formulations is described (6). Electroanalytical methods (voltammetry and polarography) were developed for the determination of PVS in tablet dosage form and biological media (7, 8). Several methods have also been reported for the determination of PVS in biological samples. These include HPLC with ultraviolet detector (9-14), laser-induced fluorescence (LIF) detector (15), liquid chromatography/tandem mass spectrometry (LC/MS/MS) (16-18) and capillary electrophoresis (CE) (19). In addition to these methods, a liquid chromatographic using atmospheric pressure chemical ionization mass spectrometry (LC/APCIMS) (20) and gas chromatographic employing chemical ionization mass spectrometry (GC/CIMS) (21-23) methods have been used for the analysis of PVS. However, mass-selective detection is cost intensive and often not available for routine analysis. Other authors have published method for plasma and urine using radioactive labeled pravastatin (24). This method requires special equipment and safety conditions. The objective of this work was to develop and validate a rapid and reliable analytical method using reversed phase-high performance liquid chromatography (RP-HPLC) for determination of PVS in tablets pharmaceutical formulations.

## EXPERIMENTAL

### Chromatographic system

Chromatographic analysis was performed on a modular HPLC system, Jasco (Japan) consisted of binary pump (PU-2089 Plus, flow rate range of 0.05-5 mL min^-1^), auto sampler (AS-2059 Plus, injection volume of 0.1-200 μL), column oven (CO-2065 Plus, temperature range of 10-80°C) and ultraviolet detector (MD-2015 Plus, 200-900 nm) operated at wavelength of 239 nm and a quartz flow cell (10 mm path and 17 μL volume). Separation was achieved on a reversed phase Tracer Extracil C8 column (250 × 4.6 mm, 5 μm particle size, Teknokroma, Spain). The mobile phase was a mixture of a 10 m*M* ammonium acetate: methanol: triethylamine (40:60:0.17 v/v/v) and was filtered and degassed by ultrasonic agitation before use. The mobile phase was prepared weekly and was delivered at a flow rate of 1.0 ml/min. Data were monitored and processed using automation system software. Peak areas were integrated automatically by computer using the Borwin-PDA Jasco software program. The injection volume was 10 μL. The system was operated at ambient temperature.

### Reagents

HPLC grade methanol and water were purchased from Merck (Darmstadt, Germany) and analytical reagent grade ammonium acetate (Merck) was used to prepare the mobile phase.

### Materials

Pravastatin sodium (446.52 g/mole), batch 200703008 was supplied by CHEMLINE Healthcare (Lugano, Switzerland), and had a purity of greater than 99% according to the compendial method.

Pravastatin tablets supplied by Elsaad Pharma (Aleppo, Syria), each tablet was labelled to contain pravastatin sodium 20 or 40 mg and pravastin tablets supplied by Rasha (Aleppo, Syria), each tablet was labelled to contain pravastatin sodium 20 or 40 mg.

### Standard Solutions

Standard stock solution of pravastatin sodium in concentration of 1 mg/mL was prepared by dissolving required amount of pravastatin sodium in mobile phase and stored at 2-8°C. The solution was stable for three weeks at least. The working standard solutions of PVS-Na (0.4-1000 μg/mL) were prepared by diluting the standard stock solution with the mobile phase.

### Calibration Curve

To construct the calibration curve five replicates (10 μL) of each standard solution were injected immediately after preparation into the column and the peak area of the chromatograms were measured. Then, the standard curves were constructed using mean peak values (Table 1).

**Table 1:** Calibration data for the standard curve of the peak area versus concentration of PVS and linear regression analysis

Parameters	Pravastatin

Optimum concentration range (μg/mL)	0.4-1000
Regression equation for the standard curves y=m x+b^a^	
Correlation coefficient (R^2^)	0.9998
Slope (m)	2.4437
Intercept (b)	1.9811
Observed drug concentration against the theoretical values	
Slope of regression line^b^	0.9999
Intercept of the regression line	0.0422
Student’s t-test^c^ (2.310)^d^	1.9650

a *y* is the peak area and *x* is the concentration in μg/mL;

b Observed vs. theoretical;

c Comparison with pharmacopoeial methods;

d Value in parenthesis is the theoretical t-value for five of degrees of freedom.

### Assay Procedure for Dosage Forms

Twenty tablets containing pravastatin sodium were weighed and finely powdered. Five accurately weighed quantities of the powder equivalent to 100 mg of PVS-Na were transferred into 100 mL separated volumetric flasks. A 90 mL mobile phase was then added to each flask and the mixture was shaken well for 5 min. Then, the volume of each mixture was adjusted to 100 mL with the mobile phase. The sample solutions were filtered and a suitable concentration was prepared by diluting the filtrates with mobile phase. Finally, 10 μL of each diluted sample was injected into the column and data were recorded. PVS concentrations in the samples were then calculated using peak data and standard curves.

## RESULTS AND DISCUSSION

### Chromatography and Selectivity

**Figure 2:**
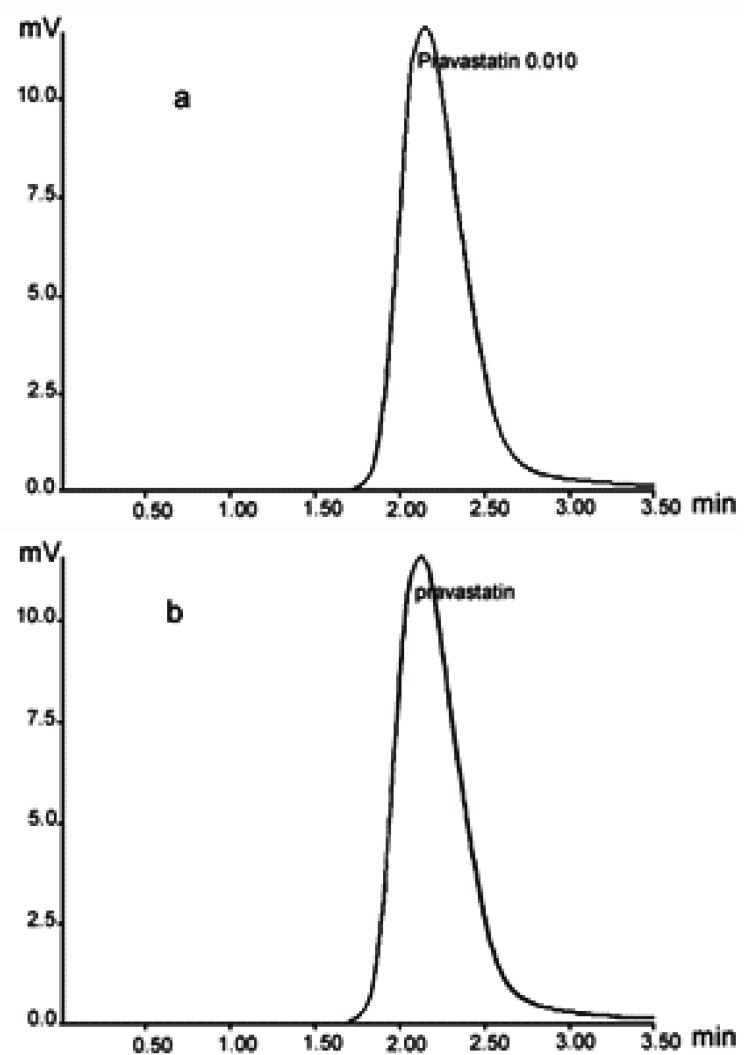
Typical chromatograms of (a) PVS standard solution (10 μg/mL) in the mobile phase and (b) PVS (10 μg/mL) in the mobile phase prepared from pravastatin 20mg tablets.

Under the chromatographic conditions employed, the typical chromatograms of PVS standard solutions and formulation samples are shown in Figure 2. The relative retention time was 2.15. The two peaks were free of interference from other components of pharmaceutical preparations (excipients), see Figure 2b.

### Calibration Curve

Calibration curve was linear over the concentration range 0.4-1000 μg/mL. Straight line for PVS was obtained, when the area of the peaks were plotted versus concentration (Table 1).

In order to establish whether the proposed method exhibits any fixed or proportional bias, a simple linear regression (25) of observed drug concentration against the theoretical values (16 points) was calculated. Student’s *t*-test (at 95% confidence level) was applied to the slope of the regression lines (Table 1) and showed that it did not differ significantly from the ideal value of unity. Hence, it can be concluded that there are no systematic differences between the determined and true concentrations over the cited ranges.

### Precision and Accuracy

The precision and accuracy of the method were evaluated by intra- (analysis of standard solutions of PVS in replicates of five in the same day) and inter-day (analysis of standard solutions of PVS in replicates of five on 3 different days from day 1 to 30 after preparation) assay variance (Table 2). The standard deviation, relative standard deviation, recovery and 95% confidence limits of different amounts tested were determined from the calibration curve, as recorded in Table 2. The accuracy of the method is indicated by the excellent recovery (100.81-103.31%) and the precision is supported by the low standard deviation.

**Table 2:** Accuracy and precision of within and between run analysis for the determination of pravastatin by high-performance liquid chromatography

Nominal concentration (μg.mL^-1^)	Assayed concentration (μg.mL^-1^)			
	Mean ± S.D	RSD%	Recovery %	Confidence limits

Intra-day (n=5)				
1.0	1.02 ± 0.03	2.94	102.00	1.02 ± 0.04
10.0	10.21 ± 0.18	1.76	102.17	10.21 ± 0.22
20.0	20.40 ± 0.35	1.71	102.04	20.40 ± 0.43
40.0	40.65 ± 0.70	1.72	101.62	40.65 ± 0.87
100.0	101.94 ± 1.66	1.62	101.94	101.94 ± 2.06
500.0	516.58 ± 4.28	0.83	103.31	516.58 ± 5.31
1000.0	1025.40 ± 1.49	0.14	102.40	1025.40 ± 1.85
Inter-day (n=5)				
1.0	1.01 ± 0.03	2.97	101.00	1.01 ± 0.04
10.0	10.13 ± 0.19	1.87	101.30	10.13 ± 0.24
20.0	20.16 ± 0.33	1.64	100.80	20.16 ± 0.41
40.0	40.65 ± 0.65	1.62	101.62	40.65 ± 0.81
100.0	100.96 ± 1.32	1.31	100.96	100.96 ± 1.64
500.0	513.80 ± 3.80	0.74	102.76	513.80 ± 4.72
1000.0	1019.88 ± 1.83	0.18	101.99	1019.88 ± 2.27

### Limits of detection (LOD) and quantification (LOQ)

The minimum level at which the investigated compound can be reliably detected (limit of detection, LOD) and quantified (limit of quantification, LOQ) were determined experimentally. Limit of detection was measured as the lowest amount of PVS that may be detected to produce a response which is significantly different from that of a blank. Limit of detection for PVS was found to be 12 ng/mL. The limit of quantification (LOQ) was determined as the lowest concentration pf PVS used in the construction of the corresponding standard curve and defined as 0.4 μg/mL.

### Application of the Assay

The validity of the proposed method for the determination of PVS was assessed by measuring drug concentration of pharmaceutical dosage forms. The results obtained with the proposed method were compared with the pharmacopoeial method (26) and are shown in Table 3. Good agreement with results obtained by the pharmacopoeial methods was observed. The proposed method is simple, rapid, accurate, highly sensitive and suitable for the routine quality control without interference from the excipients and additives such as starch, glucose, lactose and magnesium stearate.

**Table 3:** Determination of pravastatin sodium in pharmaceutical formulations by the proposed method and pharmacopoeial method

Drug	Label claim	%Found^a^ ± SD	
		Proposed method	Official method

Pravastatin	20 mg/Tab	101.20 ± 0.21	100.79 ± 0.13
		*t* =2.17	*t* =1.32
		*F* =2.61	
	40 mg/Tab	100.80 ± 0.18	101.05 ± 0.18
		*t* =1.75	*t* =1.28
		*F* =1.00	
Pravastin	20 mg/Tab	100.73 ± 0.23	99.89 ± 0.15
		*t* =1.36	*t* =1.79
		*F* =2.35	
	40 mg/Tab	101.08 ± 0.27	100.52 ± 0.21
		*t* =1.12	*t* =1.81
		*F* =1.65	

a Five independent analyses. At 95% confidence level t-value is 2.776 and F-value is 6.26.

## CONCLUSION

The proposed method for determination of pravastatin by RP-HPLC with ultraviolet detection yielded excellent recoveries and precision. The method was shown to be simple, rapid and reproducible. The validity of the developed method is well demonstrated by analysis of pravastatin tablets with different label claims. This method was found to be highly sensitive and there is no need for special column and precolumn or postcolumn treatment of sample. Moreover, the method is free from interference by common additives and excipients, suggesting applications in routine quality control.
